# Outcomes of posterior spinal fusion in AIS: A minimally invasive vs. open technique analysis

**DOI:** 10.1016/j.bas.2026.106029

**Published:** 2026-03-31

**Authors:** Mario D. Ropelato, Jacopo Vitale, Anne F. Mannion, Friederike Schömig, Jani Puhakka, Markus Loibl, Fabio Galbusera, Dezsö Jeszenszky, Tamás F. Fekete

**Affiliations:** a Spine Center, Schulthess Klinik, Zürich, Switzerland; b Institute of Sports Sciences, University of Physical Culture in Cracow, 31-571, Cracow, Poland; c Center for Musculoskeletal Surgery, Charité - Universitätsmedizin Berlin, Berlin, Germany

**Keywords:** Adolescent idiopathic scoliosis, Minimally invasive spinal surgery, Posterior spinal fusion, Surgical outcomes, COMI

## Abstract

**Introduction:**

Posterior spinal fusion is the most popular surgical treatment for adolescent idiopathic scoliosis (AIS). Recent innovations in implant designs have facilitated minimally invasive (MISS) approaches. However, these techniques have not yet achieved widespread adoption for deformity correction.

**Research question:**

This study aimed to compare surgical, radiologic, and clinical outcomes in AIS patients treated with a minimally invasive transmuscular (MINI-OPEN) approach versus the traditional open (OPEN) approach.

**Material and methods:**

This single-center retrospective study analyzed prospectively collected data from the EUROSPINE Spine Tango Registry framework. Inclusion criteria were AIS, posterior fusion, major coronal Cobb angle <80°, and 10-30 years. Complications, blood loss, operative time, length of stay (LOS), and pre- and postoperative radiographs were evaluated. Patients completed the Core Outcome Measures Index (COMI) preoperatively and at 3, 12, and 24 months.

**Results:**

Seventy-one OPEN patients were compared with 39 MINI-OPEN patients. Blood loss was significantly lower in the MINI-OPEN group (350.0 (275.0-500.0) vs 1150 (600.0-1625.0)mL), whereas operative time was significantly longer (450 (389.0-522.0) vs 326.0 (259.0-403.0)minutes). LOS was nearly half as long in the MINI-OPEN as in the OPEN group (5.0 (4.0-6.0) vs 9.0 (8.0-11.0)days). Coronal curve correction (median reduction 77% MINI-OPEN vs 75% OPEN) and COMI improvements did not differ significantly between groups.

**Discussion and conclusion:**

The MINI-OPEN approach was associated with less blood loss and a shorter hospital stay but longer operative time compared with the OPEN approach while radiologic and patient-reported outcomes were similar. These findings support MINI-OPEN as a feasible soft-tissue-sparing strategy in AIS correction.

## Highlights

• Transmuscular MINI-OPEN approach reduces blood loss vs conventional OPEN approach in posterior AIS correction.
• MINI-OPEN approach shortens hospital stay nearly by half compared with OPEN approach.
• Curve correction is equivalent between MINI-OPEN and OPEN approaches.
• Patient-reported outcomes are similar for MINI-OPEN and OPEN approaches.

## 1 Introduction

Adolescent idiopathic scoliosis (AIS) is a complex spinal deformity that affects many adolescents worldwide, with a prevalence of 0.47-5.2% (Konieczny et al., 2013). Due to its possible detrimental effects with curve progression, pain, and cosmetic considerations, surgical treatment may be warranted. The most popular surgical treatment method for AIS is posterior spinal fusion (Lonner et al., 2018). However, given its invasiveness and the extensive muscle and tissue damage it causes, minimally invasive scoliosis surgical (MISS) approaches have been developed. These aim to reduce muscle trauma, postoperative pain, blood loss, and length of hospital stay (Park et al., 2022). However, MISS presents challenges in intraoperative orientation due to the narrower approach and limited surgical overview.

Recent literature has increasingly focused on the efficacy and safety of MISS techniques in AIS (Park et al., 2022), yet less is known about their impact on outcomes that matter most to patients, such as pain, function, and overall quality of life. Patient-reported outcome measures (PROMs), such as the Core Outcome Measures Index (COMI), are essential for capturing these dimensions, offering insight into how surgery affects the patient's pain and function in their daily life and their overall well-being (Mannion et al., 2009a).

Only few previous studies have analyzed PROMs in patients with AIS undergoing MISS (Yang et al., 2020, 2021a) and even fewer have compared MISS with the open approach (Miyanji et al., 2015; Zhu et al., 2017; Yang et al., 2021b; Sarwahi et al., 2024). Findings from these studies generally indicate an improvement in PROMs following correction surgery. However, differences between the two surgical approaches are not always statistically significant, with only tendencies reported (Yang et al., 2021b) or improvements limited to specific subitems of the SRS-22 questionnaire. Additionally, follow-up periods for PROMs are often inconsistently defined, and the surgical techniques used in previous studies vary from our own - for example, the use of an O-arm (Zhu et al., 2017) or a less muscle-sparing approach in MISS, particularly concerning the rhomboid and trapezius muscles.

The present study aimed to compare surgical, radiologic, and - most importantly - clinical outcomes at defined short- and long-term follow-up intervals in AIS patients treated with MISS (i.e. MINI-OPEN) versus the traditional open approach. By evaluating surgical parameters, length of hospital stay (LOS), complications, and curve correction up to two years postoperatively, we sought to clarify the potential advantages and limitations of each technique and hence provide valuable insights for clinical decision-making and the establishment of appropriate patient expectations. We hypothesized that the muscle-sparing MINI-OPEN approach would be associated with lower blood loss, shorter LOS, and better short-term (3-month) COMI scores compared with the open approach, while maintaining radiographic curve correction.

## 2 Methods

### 2.1 Study design and subjects

This was a retrospective analysis of data collected prospectively within a single Spine Centre from 2005 to 2021, using the EUROSPINE Spine Tango Registry framework. The database, which was introduced in 2005, documents surgical and PROMs data (see later) for all patients undergoing surgery for spinal disorders in our hospital (Mannion et al., 2009b). Patients operated using either conventional open methods (OPEN) or a minimally invasive posterior transmuscular approach (MINI-OPEN) were screened. The two surgical approaches were performed during different time periods within the study interval; OPEN during 2005-2016 and MINI-OPEN during 2016-2021. The sample included all eligible patients. Inclusion criteria were: adolescent idiopathic scoliosis; posterior spinal fusion as surgical treatment; major coronal Cobb angle <80°; 10 ≤ age ≤30; for PROMs patients <12 were excluded, since per institutional policy COMI questionnaires are only sent to patients ≥12 years old. Excluded were patients with osteotomies ≥ Schwab Grade 2 (Schwab et al., 2015) and accessory osteotomies (e.g. ribs), use of navigation, no preoperative full radiographic work up, and presence of documented refusal. Ethical approval was obtained for the re-use of the registry data for this study (BASEC, 2024-02406). All patients had consented to their data being used. The study was conducted in compliance with the current national and international laws and regulations governing the use of human subjects (Declaration of Helsinki II).

### 2.2 Surgical approach

**Fig. 1:**
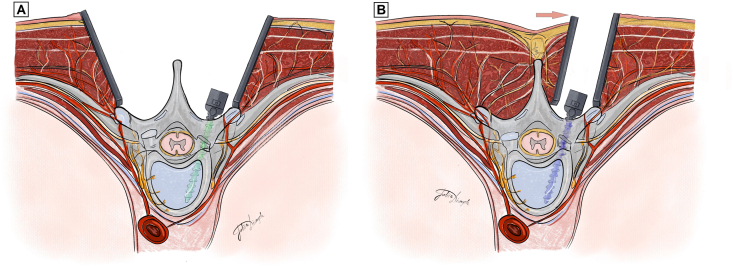
Illustration of OPEN (A) and MINI-OPEN (B) approach. The OPEN approach involves a midline skin incision followed by bilateral detachment of paraspinal musculature from the posterior elements to allow wide exposure of the fusion levels. In the MINI-OPEN approach, a midline skin incision is performed; the overlying skin and subcutaneous tissue are subsequently mobilized and shifted laterally (arrow) to enable creation of a paramedian transmuscular corridor through the deep fascia, developed sequentially on both sides. This corridor-based exposure preserves the longer paraspinal muscles (erector spinae, rhomboids, trapezius) that span multiple segments and connect the spine to the upper extremities, while selectively detaching the multifidus muscles at the fusion levels. The approach utilizes a narrow longitudinal surgical corridor aligned with the facet joints and pedicle screw trajectories, allowing adequate fusion bed preparation and complete deformity correction maneuvers (rod derotation, direct vertebral rotation, segmental correction) without the need for wide bilateral muscle detachment.

Surgery in the traditional OPEN group was performed collectively by 5 surgeons in our department using the same overall technique. The MINI-OPEN surgeries were a consecutive series performed by a single surgeon. As previously described (Sarwahi et al., 2011; de Bodman et al., 2017), the MINI-OPEN technique involves making 2 to 3 midline incisions, followed by a muscle-sparing transmuscular paramedian approach for the placement of transpedicular screws (Fig. 1). This approach strategically aims to preserve the functional anatomy of the posterior musculature, particularly the longer paraspinal muscles such as the longissimus and iliocostalis, which span multiple vertebral levels and contribute to dynamic spinal stability, especially in the mid and upper thoracic spine. Dissection is performed carefully to preserve the rhomboid and trapezius muscles by following their natural fiber orientation. At the instrumented levels, controlled detachment of the multifidus muscles is performed. This is considered anatomically justified, as the segmental function of the multifidus is effectively eliminated by the fusion process itself, regardless of surgical technique.

The technique leverages the anatomical alignment between the facet joints and the ideal pedicle screw trajectory, allowing for direct visualization and preparation of the fusion bed through the same corridor used for screw insertion. Facetectomies and decortication of the fusion site are thus performed without compromising soft tissue integrity. Pedicle screws are placed freehand, without fluoroscopic assistance, and submuscular rods are inserted through the incisions. Typically, instrumentation follows a pattern of 2–3 vertebrae in a set, leaving one vertebra between sets uninstrumented; this reflects the operating surgeon's preferred instrumentation strategy rather than a technical requirement of the MINI-OPEN exposure and does not limit the extent or distribution of fusion levels, which remains consistent with traditional open procedures.

Correction maneuvers, including rod derotation, direct vertebral rotation, and segmental translation, are executed using the same biomechanical principles as in standard open surgery.

### 2.3 Outcome measures

#### 2.3.1 Baseline characteristics

The following baseline characteristics were recorded: age; sex; Risser stage, as a measurement of skeletal maturity, using the development of the iliac crest apophysis (Hacquebord et al., 2012); Lenke type, a commonly used method of scoliosis classification that can help as a guide to treatment strategy (Hacquebord et al., 2012).

#### 2.3.2 Surgical parameters

Surgical parameters included total blood loss (mL), calculated as the total blood volume lost minus the amount retransfused from a cell saver device, and blood loss per instrumented level (mL/level), determined by dividing the total blood loss by the number of instrumented levels. Surgical time (minutes) was recorded as the total duration of the procedure (incision to wound closure), and surgical time per fused level (minutes/level) was calculated by dividing the total surgical time by the number of fused levels. Additional data recorded included the specific vertebrae instrumented, the number of fused segments (defined as the planned arthrodesis levels corresponding to the instrumented construct as documented in the operative report and postoperative radiographs), and the number of screws per vertebra. As this was a retrospective registry analysis, and postoperative CT imaging was not routinely available, arthrodesis status could not be confirmed. The length of stay (LOS, days) was calculated from the date of surgery to the date of discharge, with each day counted as a full day, even if discharge occurred in the morning. Furthermore, complications were systematically assessed for all patients during the full follow-up period of 2 years using operative, discharge, and consultation reports. Complications were grouped into 4 categories: revision surgeries, infections, neurological deficits, and internal medicine related issues.

#### 2.3.3 Imaging parameters

An experienced surgeon observer (M.R.) analyzed pre- and postoperative plain whole spine standing X-rays obtained as part of standard clinical care for major curve coronal and thoracic kyphosis Cobb (T2-12, T5-12) angles according to the methods described by (Carman et al., 1990; Kim et al., 2010). Excellent inter- and intra-rater reliability have previously been reported for these methods (Gstoettner et al., 2007; Modi et al., 2009; Wu et al., 2014). Major curve angles were recorded directly postoperatively and at around 3 months, 1 year and 2 years postoperatively. Preoperative and 3-month postoperative data were compared to assess short-term changes as a result of the surgery. These radiographic parameters were selected as the predefined measures of structural correction for this study.

#### 2.3.4 COMI

Patients completed the Core Outcome Measures Index (COMI) (Deyo et al., 1998; Mannion et al., 2005; Ferrer et al., 2006) preoperatively and at 3, 12, and 24 months postoperatively. COMI is a validated PROM used to assess the key outcomes that are important for patients with back disorders: back and leg/buttock pain (each measured on a 0-10 numeric rating scale), and back-related function, symptom-specific well-being, quality of life, social and work disability (each measured on a 0-5 scale). All items refer to “the last week”, except disability (“the last 4 weeks”). The final COMI score results in a value ranging from 0 (best status) to 10 (worst status) (Mannion et al., 2009b, 2015; Genevay et al., 2012) and the minimal clinically important change score (MCIC) for individual improvement is reported to be 2.2 points (Mannion et al., 2009). Several language versions are available, including German, English, French, and Italian (covering the main languages spoken in Switzerland). Levels of satisfaction with care (measured using a 5-point Likert scale ranging from "very satisfied" to "very dissatisfied") and the global treatment outcome (GTO, measured using a 5-point Likert scale ranging from operation "helped a lot" to "made things worse") were assessed. GTO and satisfaction responses were dichotomized to facilitate clinical interpretation. For GTO, responses of 1 or 2 ('Helped a lot' or 'Helped') were classified as a 'good outcome', while responses of 3 to 5 ('Helped only little', 'Didn't help', or 'Made things much worse') were grouped as 'poor outcome'. Similarly, for satisfaction, responses of 1 or 2 were classified as 'Satisfied', while responses of 3 to 5 were classified as 'Not satisfied'.

### 2.4 Statistical analysis

As this was a retrospective cohort study, multiple clinically relevant outcomes were assessed and no single primary outcome was predefined. Descriptive statistics were used to summarize the baseline characteristics of the study population. Categorical variables are presented as frequencies and percentages, and continuous variables as mean ± standard deviation or median and interquartile range, as appropriate. Normality of continuous variables was assessed using the Shapiro–Wilk test.

For between-group comparisons, normally distributed continuous variables were analyzed using unpaired Student's t-tests and non-normally distributed continuous variables using the Mann–Whitney test. Hodges–Lehmann estimates of the between-group difference with 95% confidence intervals were additionally reported. Categorical variables were compared using the Chi-square test or Fisher's exact test, as appropriate.

#### 2.4.1 Baseline differences and surgical parameters

Baseline between-group comparisons were performed according to the distribution and type of variable as described above. Differences between groups in surgical parameters were analyzed according to the distribution of the respective variable.

#### 2.4.2 X-ray parameters, COMI, and complications

To test the hypothesis of superior 3-month PROMs in the MINI-OPEN group, we calculated change-scores (3-month minus baseline) for COMI. The delta values were normally distributed and therefore compared between the MINI-OPEN and OPEN groups using an independent *t*-test. A two-way repeated-measures analysis of variance (ANOVA) was used to assess radiographic outcomes, with time (PRE vs POST) as the within-subject factor and group (MINI-OPEN vs OPEN) as the between-subject factor, for the major Cobb curve, T2–T12 kyphosis, and T5–T12 kyphosis. Bonferroni correction was applied for multiple comparisons. Due to incomplete follow-up data, COMI scores, as well as COMI subdomain, satisfaction, and GTO scores, were analyzed using a mixed-effects model with REML estimation in GraphPad Prism, under a missing-at-random assumption. The models included fixed effects for group, time, and group × time interaction with a random intercept for subject to account for repeated measurements over time. Post hoc pairwise comparisons were adjusted using Bonferroni correction. A Chi-square test was used to evaluate differences in the occurrence of the different categories of complication. The level of significance was set at p < 0.05. Statistical analysis was carried out using GraphPad Prism (version 9.00; GraphPad Software, San Diego, CA).

## 3 Results

### 3.1 Subjects

**Fig. 2:**
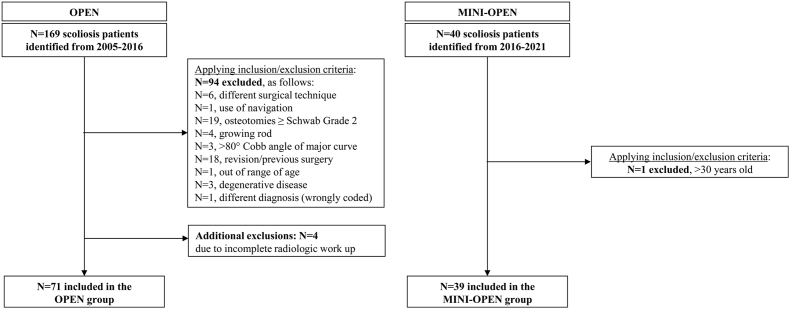
Flowchart of inclusion and exclusion criteria for the MINI-OPEN and OPEN group.

Searching the term "scoliosis" in the surgery and diagnosis fields in our institutional database, a total of 169 patients operated between 2005 and 2016 were identified for the OPEN group. After applying the inclusion and exclusion criteria, 75 patients met the eligibility requirements (Fig. 2). Of these, four patients were excluded due to incomplete preoperative radiologic workup, resulting in a final cohort of 71 patients in the OPEN group. These patients were operated on by five different surgeons. The MINI-OPEN group consisted of a consecutive series of AIS cases operated on between 2016 and 2021 by a single surgeon, who was one of the five surgeons performing surgeries in the OPEN group. A total of 40 patients were identified, with one exclusion (>30 years old), resulting in a final sample size of n = 39.

The two study groups were similar at baseline, with a gender ratio M/F ∼ 1/5 in both groups (p > 0.99), BMI ≈20 kg/m^2^ in both groups (p = 0.79), major curve Cobb angle of 55.8 ± 8.5° MINI-OPEN vs 58.1 ± 8.8° OPEN (p = 0.21), Risser stage (p = 0.70) and Lenke type (p = 0.51), except for age, which was 15.5 ± 2.7 years MINI-OPEN vs 17.1 ± 3.5 years OPEN (p = 0.01). Baseline characteristics are presented in Table 1.

**Table 1:** Baseline characteristics.

Baseline characteristics	MINI OPEN *(n = 39)*	OPEN *(n = 71)*	p-value
**Age***(years)*	15.5 ± 2.7	17.1 ± 3.5	0.01
**Sex ratio***(% males)*	17.9%	18.3%	>0.99
**BMI***(kg/m*^*2*^*)*	20.5 ± 3.7	20.7 ± 3.3	0.79
**Lenke curve**			
1	25 (64.1%)	39 (54.9%)	0.51
2	6 (15.4%)	8 (11.3%)	
3	1 (2.6%)	9 (12.7%)	
4	0 (0.0%)	1 (1.4%)	
5	4 (10.2%)	9 (12.7%)	
6	3 (7.7%)	5 (7.0%)	
**Risser stage**			
0	3 (7.7%)	3 (4.2%)	0.70
1	4 (10.2%)	7 (9.9%)	
2	4 (10.2%)	8 (11.3%)	
3	7 (17.9%)	7 (9.9%)	
4	10 (25.8%)	17 (23.9%)	
5	11 (28.2%)	29 (40.8%)	
**Major Cobb curve***(degrees)*	55.8 ± 8.5	58.1 ± 8.8	0.21
**T2 – T12 kyphosis***(degrees)*	29.9 ± 12.4	30.5 ± 13.6	0.86
**T5 – T12 kyphosis***(degrees)*	21.7 ± 10.2	20.7 ± 11.7	0.66
**COMI***(points)∗*	3.9 ± 2.3	3.4 ± 2.2	0.85

Results are reported as mean ± SD. *Abbreviations*: BMI, Body Mass Index; COMI, Core Outcome Measures Index. ∗Available in 30/39 MINI-OPEN patients and 71/71 OPEN patients.

### 3.2 Surgical parameters and LOS

Surgical outcomes are presented in Table 2. Median LOS was significantly lower in the MINI-OPEN group than in the OPEN group (5.0 vs 9.0 days, respectively, p < 0.0001), with a Hodges–Lehmann estimated between-group difference of 4 days (95% CI, 3 to 5), as was median blood loss (350.0 vs 1150 mL, respectively, p < 0.0001) with a difference of 700 mL (95% CI, 400 to 900), whereas median surgical time was longer (450 vs 326.0 min, respectively, p < 0.0001), with a difference of 125 min (95% CI, 86 to 168).

**Table 2:** Surgical parameters.

Surgical parameters	MINI OPEN *(n = 39)*	OPEN *(n = 71)*	p-value
**Blood loss***(mL)*	350.0 (275.0-500.0)	1150 (600.0-1625.0)	**<0.0001**
**Blood loss per level***(mL)*	43.0 (34.0-63.9)	125.0 (82.25-182.3)	**<0.0001**
**Blood loss per screw***(mL)*	25.0 (20.0-36.0)	61.0 (38.0-146.0)	**<0.0001**
**Surgical time***(min)*	450 (389.0-522.0)	326.0 (259.0-403.0)	**<0.0001**
**Surgical time per level***(min)*	53.0 (47.0-59.0)	38.0 (31.0-46.0)	**<0.0001**
**Surgical time per screw***(min)*	30.0 (28.0-33.0)	17.0 (15.0-21.0)	**<0.0001**
**LOS***(days)*	5.0 (4.0-6.0)	9.0 (8.0-11.0)	**<0.0001**
**Screws***(n)*	14 (12-18)	19 (15-22)	**<0.0001**
**Instrumented vertebra***(n)*	8 (6-9)	10 (8-11)	**<0.0001**
**Fused segments***(n)*	9 (7-10)	9 (7-10)	=0.54
**Screws per vertebra***(n)*	1.6 (1.5-1.6)	2.0 (1.9-2.0)	**<0.0001**

Results are reported as median (IQR). The differences were evaluated with the Mann-Whitney test for each variable. *Abbreviations*: LOS, length of stay.

### 3.3 Imaging parameters

**Fig. 3:**
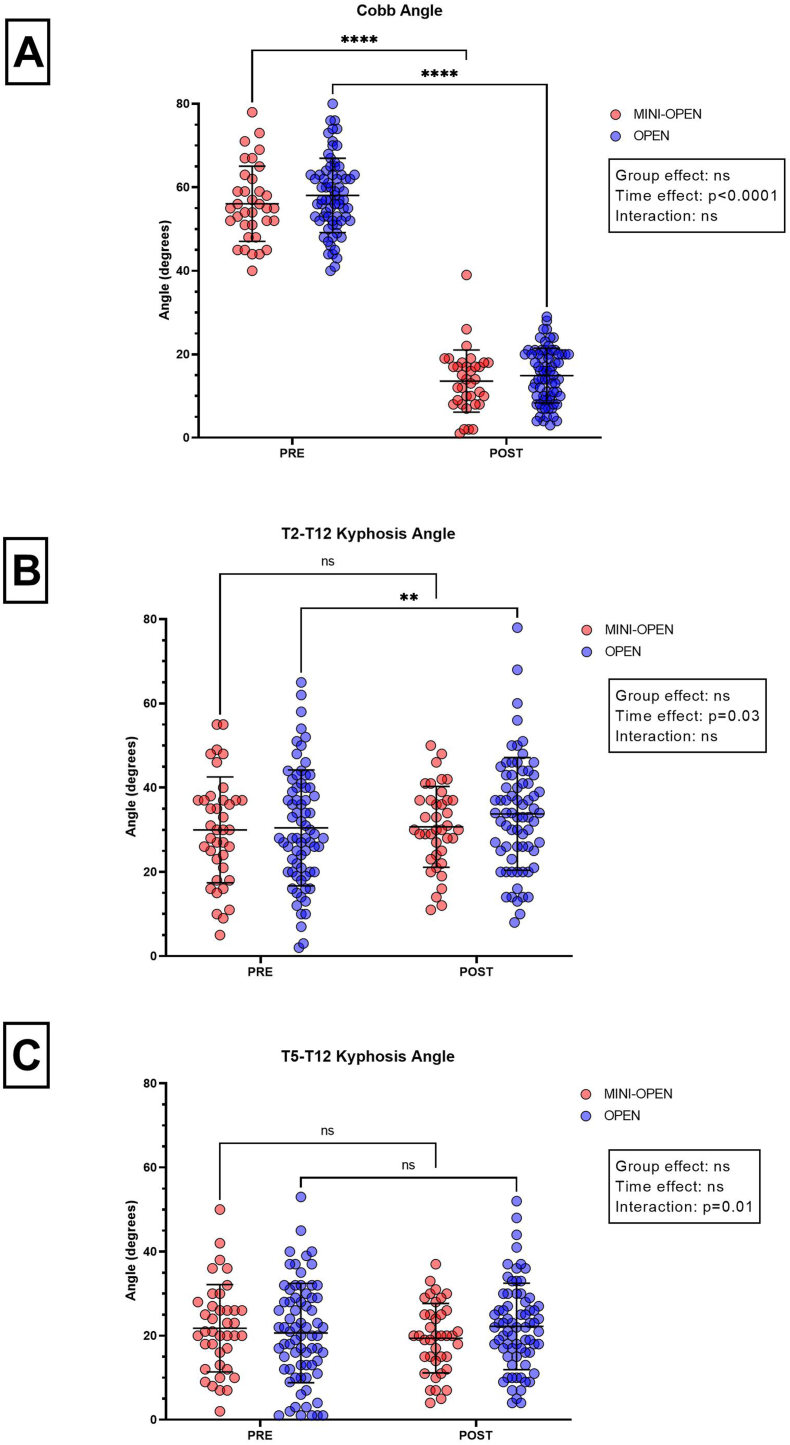
Individual data plots (n = 39 for MINI-OPEN, red circles and n = 71 for OPEN, blue circles) and mean ± SD (black lines) of major Cobb curve (panel A), T2-T12 kyphosis (panel B), and T5-T12 kyphosis (panel C) evaluated at PRE and POST (3 months postop.). Legend: ∗∗p < 0.01; ∗∗∗∗p < 0.0001.

Raw data and mean ± SD for major curve Cobb angle, T2-T12 kyphosis, and T5-T12 kyphosis angles are shown in Fig. 3 (panels a, b, and c, respectively) while Table 3 shows mean ± SD values and the results of the 2-way ANOVA. No statistically significant difference in major Cobb curve correction was observed between the two groups with delta values of 42.2 and 43.2° for MINI-OPEN and OPEN, representing a median reduction of 77% and 75% respectively, with no statistically significant between-group difference (Mann–Whitney, p = 0.272; Hodges–Lehmann difference, −2%; 95% CI, −7 to 2). The T2-T12 kyphosis angle increased significantly at POST (3 months postoperatively) only in the OPEN group (+3.4°, p = 0.006). Although no significant main effects were found for time or group in the T5–T12 kyphosis angle, a significant group × time interaction (p = 0.01) was detected. This interaction reflected a divergent pattern of change between groups, with the MINI-OPEN group showing a decrease of 2.4°, while the OPEN group exhibited an increase of 1.5° over time. Correction of major curve did not change significantly from 3 months to 2 years postoperatively and the groups did not differ in this respect (MINI-OPEN +1.9° and OPEN +0.8°; p = 0.78).

**Table 3:** Major curve Cobb angle, T2-T12 kyphosis, and T5-T12 kyphosis preoperatively (PRE) and postoperatively (POST) in MINI-OPEN and OPEN groups.

	PRE		POST		Interaction	Group Effect	Time Effect	Contrasts
	MINI-OPEN *(n = 35)*	OPEN *(n = 71)*	MINI-OPEN *(n = 35)*	OPEN *(n = 71)*				
**Major curve Cobb angle (degrees)**	55.8 ± 8.8	58.1 ± 8.8	13.6 ± 7.3	14.9 ± 6.5	ns	ns	p < 0.0001	MINI-OPEN: POST < PRE (p < 0.0001); OPEN: POST < PRE (p < 0.0001)
**T2-T12 kyphosis (degrees)**	29.9 ± 12.4	30.5 ± 13.6	30.7 ± 9.4	33.9 ± 13.2	ns	ns	p = 0.03	MINI-OPEN: ns OPEN: POST > PRE (p = 0.006)
**T5-T12 kyphosis (degrees)**	21.7 ± 10.2	20.7 ± 11.7	19.3 ± 8.1	22.2 ± 10.2	p = 0.01	ns	ns	MINI-OPEN: ns OPEN: ns

Results are reported as mean ± SD. Abbreviations: PRE: baseline evaluation; POST: 3-months evaluation; ns, no statistical differences.

Also, major curve Cobb angle at 2 years did not differ statistically between groups (MINI-OPEN: 15.2 ± 6.7° vs OPEN: 15.3 ± 6.3°, p = 0.91).

### 3.4 COMI, satisfaction and GTO

In the OPEN group, all patients completed a baseline COMI. In the MINI-OPEN group, COMI baseline data were missing for 9 (23.1%) patients, four of whom were under 12 years old. Since these four patients had not received a COMI questionnaire to complete due to our institutional policy (sending only to patients ≥12 years old), they did not contribute any COMI data and were therefore excluded from the COMI data analysis. The remaining patients with missing baseline COMI could still contribute available postoperative COMI data to the longitudinal mixed-effects analysis, whereas analyses requiring baseline values were necessarily limited to patients with available baseline COMI. Missing postoperative patient-reported outcome data were mainly attributable to non-returned follow-up questionnaires. Missingness was often intermittent rather than monotonic, with some patients missing a single follow-up assessment but providing data at later timepoints. COMI completeness relative to the full cohort was 76.9% at baseline and 74.4%, 84.6%, and 74.4% at 3, 12, and 24 months, respectively, in the MINI-OPEN group; corresponding values in the OPEN group were 100%, 95.8%, 93.0%, and 91.6% (Table 4).

**Table 4:** COMI scores preoperatively and at 3, 12, and 24 months follow-up in MINI-OPEN and OPEN groups.

		PRE	3mo	12mo	24mo	Interaction	Group Effect	Time Effect	Contrasts
**COMI**	**MINI-OPEN***(n = 39)*	3.9 ± 2.3 (n = 30, 76.9%)	2.5 ± 2.2 (n = 29, 74.4%)	1.4 ± 1.5 (n = 33, 84.6%)	1.8 ± 1.7 (n = 29, 74.4%)	ns	ns	P < 0.0001	**MINI-OPEN:** PRE>3mo (p = 0.008); PRE>12mo (p < 0.0001); PRE>24mo (p < 0.0001); 3mo > 12mo (p = 0.006)
	**OPEN***(n = 71)*	3.4 ± 2.2 (n = 71, 100%)	2.7 ± 2.2 (n = 68, 95.8%)	1.8 ± 1.8 (n = 66, 93.0%)	1.7 ± 1.6 (n = 65, 91.6%)				**OPEN** PRE>3mo (p = 0.03); PRE>12mo (p < 0.0001); PRE>24mo (p < 0.0001); 3mo > 12mo (p = 0.0012); 3mo > 24mo (p = 0.0009)

Results are reported as mean ± SD. Percentages indicate follow-up completeness relative to the full group size. Abbreviations: PRE: baseline evaluation; 3mo: 3-months evaluation; 12mo: 12-months evaluation; 24mo: 24-months evaluation; ns: no statistical differences.

**Fig. 4:**
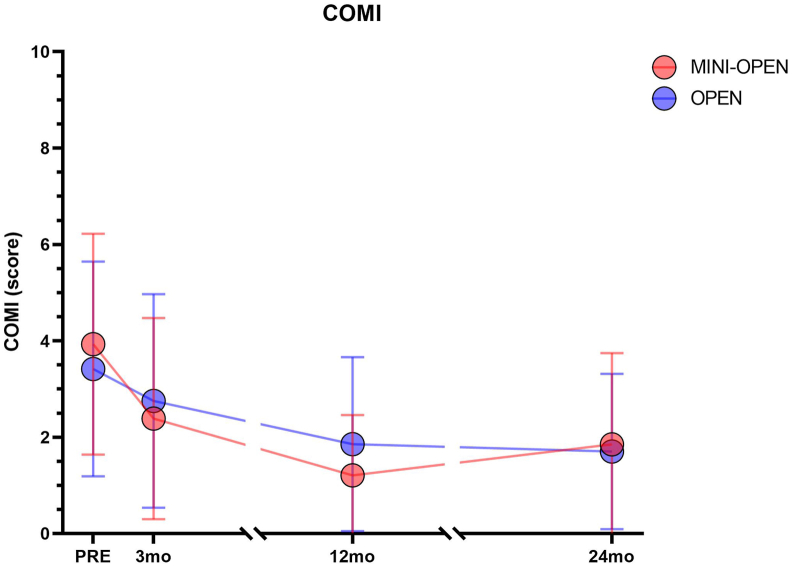
Mean ± SD of COMI scores for MINI-OPEN (red) and OPEN (blue) at 0, 3, 12 and 24 months. For graphical clarity, all significant differences are not reported here but are detailed in Table 4.

Both the MINI-OPEN and OPEN groups showed significant improvements in COMI scores over time, with no statistically significant difference between the groups (Fig. 4). In the MINI-OPEN group, the mean score decreased from 3.9 ± 2.3 at baseline to 2.5 ± 2.2 at 3 months (p = 0.008), with a further reduction to 1.4 ± 1.5 at 12 months (p < 0.0001). A slight increase was noted at 24 months (to 1.8 ± 1.7), but it remained significantly lower than at baseline. The OPEN group improved from 3.4 ± 2.2 at baseline to 2.7 ± 2.2 at 3 months (p = 0.03), then to 1.8 ± 1.8 at 12 months, and 1.7 ± 1.6 at 24 months (both p < 0.0001), showing a stable improvement pattern. Improvement in COMI from preoperative assessment to 3 months postoperatively did not differ significantly between groups (MINI-OPEN: mean delta COMI -1.412; traditional: -0.699). The mean difference was 0.713 points (95% CI -0.449 to 1.875; Welch's *t*-test: t = 1.244, df = 36.55, p = 0.221).

**Fig. 5:**
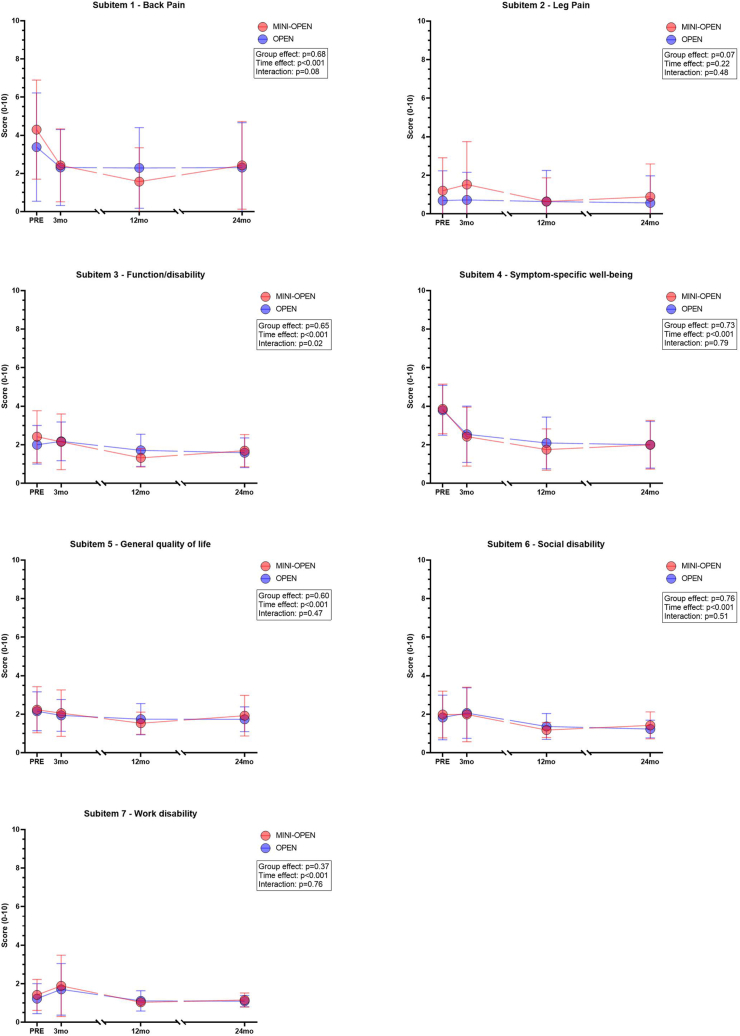
Mean ± SD of the seven COMI subitems for MINI-OPEN (red) and OPEN (blue) at 3, 12, and 24 months.

The COMI sub-domain scores are shown in Fig. 5. The mixed-effects model revealed a significant effect of time (p < 0.001) for all items other than 'leg pain', indicating an overall improvement in both groups over the follow-up period. There were no significant differences between the MINI-OPEN and OPEN groups for any item (p-values ranging from 0.07 to 0.76), at the individual timepoints. However, a significant group × time interaction was observed for 'function' (p = 0.02), indicating a different pattern of improvement over time between groups for this domain: while the MINI-OPEN group showed a gradual and sustained functional improvement over time, the OPEN group experienced an initial slight worsening (higher scores), followed by improvement. There were no significant interaction effects for any of the other individual COMI domains (p-values ≥0.08), suggesting similar trajectories of change over time in both groups.

Satisfaction and GTO scores remained consistent over time with no statistically significant differences between the MINI-OPEN and OPEN groups. In the MINI-OPEN group, 100% of patients reported a “good” outcome, defined as 'operation helped' or 'operation helped a lot', at both 3 and 12 months postoperatively. At 24 months, this proportion remained high at 96.6%, with only 1 patient (3.4%) reporting a poor outcome ('Helped only little'). In the OPEN group, 91.2% of patients (62/68) reported a “good” outcome at 3 months, increasing to 93.9% (62/66) at 12 months and remaining stable at 92.3% (60/65) at 24 months. Further, patient satisfaction rates remained high across both groups throughout the follow-up period. In the MINI-OPEN group, 27 of 29 patients (93.1%) reported being very satisfied/satisfied with their care at 3 months, 32 of 33 (97.0%) at 12 months, and 27 of 29 patients (93.1%) at 24 months. Similarly, in the OPEN group, 67 of 68 patients (95.0%) reported being very satisfied/satisfied with their care at 3 months, 64 of 66 (97.0%) at 12 months, and 61 of 65 (95.8%) at 24 months.

### 3.5 Complications

No significant differences were detected between the groups in the occurrence of complications (Table 5). Repeat surgery was performed in two cases in the OPEN group: one for insufficient proximal correction requiring construct extension and one for painful screw heads, leading to the removal of two screws 1.5 years after the index surgery. In the MINI-OPEN group, one patient required revision surgery for screw loosening and pullout, and another for painful screw heads. Additionally, in the MINI-OPEN group, one patient experienced increased lateral deviation of the C7 coronal plumb line and one patient showed progression of the thoracic, non-instrumented curve (adding-on). Both cases were treated conservatively.

**Table 5:** Occurrence of complications in MINI-OPEN and OPEN groups occurring at any timepoint during the 2-year follow-up of MINI-OPEN and OPEN groups.

Complications	MINI-OPEN (n = 39)	OPEN (n = 71)	p-value
Revisions	2 (5.1%)	3 (4.2%)	>0.99
Surgical site infections	0 (0%)	2 (2.8%)	0.54
Temporary neurological deficit	0 (0%)	2 (2.8%)	0.54
Internal medicine related complications (pyelonephritis, pleural effusion, decubitus, EC/FFP substitution)	1 (2.6%)	6 (8.4%)	0.42

Data are reported as absolute (count) and relative (percentage) values. EC: erythrocyte concentrate; FFP: fresh frozen plasma.

In the OPEN group, one patient had a conservatively managed superficial infection, and another developed a deep wound infection requiring surgical treatment (without implant removal) one month postoperatively. No infections were observed in the MINI-OPEN group. No permanent neurological deficits occurred in either group. However, two patients in the OPEN group reported temporary sensory deficits.

Other complications included one case of pleural effusion, one case of pyelonephritis, and four patients requiring erythrocyte concentrate or fresh frozen plasma transfusions in the OPEN group. In the MINI-OPEN group, one case of grade 1 decubitus ulcer was noted.

Interestingly, when the first 20 patients operated on in the MINI-OPEN group were compared with the remaining 19 patients, the more "recent" patients showed a trend toward improved surgical metrics, including lower blood loss (−147 mL), shorter surgical time (−68 min), fewer instrumented vertebrae, fewer screws, and shorter fused segments. However, none of these differences reached statistical significance.

## 4 Discussion

With our study, we aimed to assess the outcomes associated with the muscle-sparing MINI-OPEN approach in comparison to the traditional open approach for the correction of AIS. We observed lower blood loss and a significantly shorter LOS with the MINI-OPEN approach, but no significant group differences in COMI scores at the short and medium term follow-ups. As such, the hypothesis of superior short-term outcomes with the MINI-OPEN approach was not supported, although there was a suggestion for a different pattern of recovery for 'function'. The amount of correction was comparable between the two approaches.

Our study population's baseline characteristics were consistent with those reported in the literature (Miyanji et al., 2015; Zhu et al., 2017; Miyanji et al., 2013; Sarwahi et al., 2016; Sarwahi et al., 2021; Urbanski et al., 2019; de Bodman et al., 2020), with no significant differences between the groups except for age, which was significantly lower in the MINI-OPEN group than the OPEN group, albeit with similar Risser stage scores.

In our cohort, estimated blood loss was significantly lower in the MINI-OPEN group than in the OPEN group, which aligns with findings from most previous studies (Miyanji et al., 2013, 2015; Zhu et al., 2017; Sarwahi et al., 2016, 2021; Urbanski et al., 2019). Reported mean blood loss ranges between 138 ml and 600 ml for the MINI-OPEN approach and from 450 ml to 800 ml for the OPEN approach. Our results for the MINI-OPEN approach fall within this range, whereas those for the OPEN approach appear higher than those previously reported. This discrepancy may be attributed to differences in surgical techniques, potentially leading to longer operating times, as discussed below. Variations in the use of antithrombotic agents and the cell saver technique might also contribute to these differences. In addition, evolving anesthetic management and perioperative blood conservation strategies over time may have influenced intraoperative blood loss independently of the surgical approach.

The LOS in the MINI-OPEN group was almost half that of the OPEN group. Urbanski et al. reported a similar difference in LOS (7 vs 3 days) (Urbanski et al., 2019) while other studies have shown no or smaller differences (Miyanji et al., 2013, 2015; Zhu et al., 2017; Sarwahi et al., 2016, 2021). These discrepancies may be influenced by variations in discharge policies and healthcare reimbursement systems. In our study, patients were discharged once they had manageable pain, normal ambulation (including stairs), and dry wounds. The observed reductions in blood loss and LOS are clinically relevant and would be consistent with a reduced invasiveness of the MINI-OPEN approach, potentially facilitating faster recovery, wound healing, and mobilization; however, LOS is strongly influenced by institutional discharge policies and evolving perioperative care pathways, and therefore cannot be attributed solely to the surgical exposure technique.

Operative time was significantly longer in the MINI-OPEN group compared with the OPEN group. The literature reports operative times ranging from 252 to 475 min and 192-350 min, respectively (Miyanji et al., 2013, 2015; Zhu et al., 2017; Sarwahi et al., 2016). The longer operative time in the MINI-OPEN group is likely due to the more challenging orientation, narrower surgical corridor, and the inability to work on both sides simultaneously, unlike in the OPEN approach. The increased operative time represents an important consideration, as prolonged operating room occupancy may have organizational and economic implications that must be weighed against potential perioperative advantages. Only one study reported a similar operating time for both approaches (279 vs 259 min for MINI-OPEN and OPEN groups, respectively) (Sarwahi et al., 2021), which may be attributed to a larger sample size, including cases performed later in the learning curve of the newer MISS technique.

Importantly, the MINI-OPEN technique affects only the approach, not the length of the construct. While the number of fused segments was similar for the two groups, fewer screws were used in the MINI-OPEN group, as not all vertebrae were instrumented, leaving one or two vertebrae out of the construct, which reflects surgeon-specific strategies rather than the exposure alone. Nonetheless, the MINI-OPEN approach still allows for posterior fusion of all levels by performing facetectomies on all levels even without instrumenting all vertebral bodies. This difference in screw density may also have influenced blood loss, operative time, and aspects of correction strategy independently of the surgical exposure. The present comparison therefore reflects a combined surgical concept rather than the surgical approach in isolation. Compared to our results, previous studies have reported similar instrumentation lengths for both approaches with a trend toward slightly longer constructs in the OPEN approach (range of 5.7-13.0 fused segments in the OPEN groups) (Miyanji et al., 2015; Zhu et al., 2017; Sarwahi et al., 2016, 2021).

In the present study, no statistically significant difference in major curve Cobb angle correction was observed between the two approaches, and correction was maintained after 2 years of follow up. These results indicate that the MINI-OPEN approach does not contravene the surgical AIS treatment principles. The range of correction reported in the literature is 64.3%-79.2% for the MINI-OPEN and 67.8%-84.8% for the OPEN approach (Miyanji et al., 2015; Zhu et al., 2017; Sarwahi et al., 2016, 2021). Our results align with these findings. Direct comparisons of both approaches generally report similar correction rates (Fiore et al., 2022), except for two studies where correction was lower in the MINI-OPEN than in the OPEN approach (58.1 vs 67.9%, respectively ()Miyanji et al., 2015), and 68.3 vs 78.3%, respectively (Miyanji et al., 2015; Urbanski et al., 2019). These differences might have been related to the stage on the MINI-OPEN learning curve at which the two procedures were compared.

Clinical impact from the patients' perspective was assessed using COMI scores at 3 months, 1 year, and 2 years follow-up. Both groups had a significant reduction in COMI score, which was sustained over the two-year follow-up period (Table 4). However, there were no significant differences between the two groups, meaning we had to reject the hypothesis that the MINI-OPEN group would experience superior short-term (3-month) outcomes. This suggests that while a muscle-preserving concept is inherent to the MINI-OPEN technique, its measurable clinical impact remains difficult to quantify, particularly as young AIS patients generally recover well and rapidly anyway. Further research should investigate very early patient-reported outcomes and longer-term follow-up, including muscle atrophy/quality and functional assessments.

Patient-reported outcomes in AIS surgery are most commonly evaluated using the SRS-22 questionnaire. Direct comparison of our COMI-based findings with SRS-22 results reported in the literature is limited due to heterogeneity in surgical techniques and inconsistently specified follow-up intervals across studies (Yang et al., 2020, 2021a; Miyanji et al., 2015; Zhu et al., 2017; Sarwahi et al., 2024; Yang et al., 2020, 2021a; Miyanji et al., 2015; Zhu et al., 2017; Sarwahi et al., 2024). Sarwahi et al. reported no significant differences between groups for immediate postoperative pain scores but did not assess other PROMs (Sarwahi et al., 2016). A more recent study found less pain at 5-6 months' follow up in the MINI-OPEN group, while the other SRS-22 domains showed no group differences (Sarwahi et al., 2024). Zhu et al. reported less pain and better self-image for the MINI-OPEN approach (with at least 2 years' follow up) (Zhu et al., 2017). However, they used an O-ARM, so techniques cannot be compared directly (Zhu et al., 2017). While Yang et al. reported a tendency for better SRS-22 scores for self-image, mental health, and satisfaction in the OPEN group than the MINI-OPEN group, the differences were not statistically significant (Yang et al., 2021b). Miyanji et al. found no clinically significant difference in SRS-22 scores between the two approaches at 2 years’ follow up (Miyanji et al., 2015). AIS patients do not typically experience pain to any greater degree than does the general adolescent population, where back pain is fairly common (An et al., 2023). However, in subsets of patients with preoperative pain, significant improvements in pain scores have been reported after surgical curve correction in general (An et al., 2023).

Although back pain was improved and both satisfaction and GTO reflected favorable outcomes in our groups, analysis of the COMI subitems did not identify any singular domain responsible for the above-reported overall improvement in COMI, nor were there any differences between the two groups for the domain scores at any timepoint. The significant group × time interaction for 'function' might suggest a slightly different pattern of recovery in the two groups. However, this might also be influenced by the small difference at baseline and cannot be interpreted as an overall different trend for faster recovery in the MINI-OPEN.

Regarding complications, there were no significant differences found between the two groups comparing revision rates, infections, neurologic impairment, or internal medicine-related complications, although our study was not designed or sufficiently powered to detect statistically significant differences in any of these outcomes. Previous reports have found a trend towards fewer complications for MINI-OPEN than for OPEN groups (Fiore et al., 2022).

### 4.1 Strengths and limitations

Although this was a retrospective analysis, data were collected prospectively within the in-house registry that is based on the well-established EUROSPINE Spine Tango registry system with its standardized documentation forms. This ensured consistency in data collection and allowed for a comprehensive comparison of two surgical approaches in a real-world clinical setting. The OPEN group surgeries were performed by multiple surgeons, while all MINI-OPEN procedures were performed by a single surgeon. This may have introduced variability in surgical technique and outcomes, as well as potential bias for the MINI-OPEN approach. The MINI-OPEN group included cases performed during the surgeon's learning curve for the minimally invasive technique, which could have influenced outcomes such as operative time and complications, and hence potentially also patient-reported outcomes; later improvements in these parameters may not be fully captured in this analysis. A potential bias could stem from comparing two consecutive groups, with the MINI-OPEN patients being the most recently operated cases. As this represents a historical cohort comparison between two treatment eras, changes in perioperative management, anesthesia protocols, and institutional discharge pathways may have influenced outcomes independently of the surgical approach. Due to the limited sample size and the structural association between treatment era, surgeon, and surgical approach, multivariable adjustment would be unlikely to fully resolve these sources of confounding. However, our analysis demonstrated that the baseline characteristics were similar between the groups, and the follow-up rates were excellent for both MINI-OPEN and OPEN groups. This ensures reliable and representative outcome data, particularly for patient-reported measures like COMI, strengthening the validity of the conclusions drawn from the study. Nevertheless, the non-concurrent design does not allow full separation of surgical approach effects from temporal improvements in perioperative management or discharge practices. The sample size in the current study was limited, but included all available patients for the MINI-OPEN group. With group sizes of approximately n = 40 for the MINI-OPEN group and n = 70 for the OPEN group at 3 months' follow-up, the study would have had sufficient statistical power (80%) in a two-tailed *t*-test to detect a moderate effect size (corresponding to a 1.12-point (SD, 2.0) greater reduction in the MINI-OPEN group; Cohen's d = 0.56), if such a difference existed. However, the missing COMIs at baseline and the lack of COMIs for the younger patients in the MINI-OPEN group reduced the size of the MINI-OPEN group and resulted in the analyses being slightly underpowered. Lastly, while PROMs such as COMI were evaluated, the study does not include objective imaging or functional assessments of paraspinal musculature; therefore, the muscle-sparing effect remains theoretical within the context of this analysis.

## 5 Conclusion

The MINI-OPEN approach was not associated with a reduced degree of deformity correction in comparison with the OPEN approach. It was, however, associated with lower estimated blood loss and shorter LOS, although these findings likely reflect multiple contributing factors, and cannot necessarily be attributed to the muscle preserving nature of the MINI-OPEN approach. At the same time, operative time was longer in the MINI-OPEN group. Despite these differences in short-term surgical outcomes, PROMs did not differ significantly between the two approaches.

Radiologic correction appeared to be maintained over the two-year follow-up period, suggesting that a soft-tissue-sparing approach may be achievable without compromising deformity correction goals.

Further studies incorporating imaging and functional assessments of back muscle function will be essential to determine whether the muscle-sparing approach translates into superior long-term outcomes.

## Declaration of generative AI and AI-assisted technologies in the manuscript preparation process

During the preparation of this work, the authors used ChatGPT (OpenAI) to assist with language editing and improvement of phrasing. After using this tool, the authors reviewed and edited the content as needed and take full responsibility for the content of the published article.

## Declaration of competing interest

The authors declare that they have no known competing financial interests or personal relationships that could have appeared to influence the work reported in this paper.
